# Dynamic Human Error Assessment in Emergency Using Fuzzy Bayesian CREAM

**DOI:** 10.34172/jrhs.2020.03

**Published:** 2020-02-16

**Authors:** Marzieh Abbassinia, Omid Kalatpour, Majid Motamedzade, Alireza Soltanian, Iraj Mohammadfam

**Affiliations:** ^1^Center of Excellence for Occupational Health, Occupational Health and Safety Research Center, School of Public Health, Hamadan University of Medical Sciences, Hamadan, Iran; ^2^Department of Ergonomics, School of Public Health, Hamadan University of Medical Sciences, Hamadan, Iran; ^3^Modeling of Noncommunicable Diseases Research Center, Hamadan University of Medical Sciences, Hamadan, Iran; ^4^Department of Biostatistics, School of Public Health, Hamadan University of Medical Sciences, Hamadan, Iran

**Keywords:** Human error, Emergency management, Fuzzy Bayesian CREAM

## Abstract

**Background:** Human error is one of the major causes of accidents in the petrochemical industry. Under critical situation, human error is affected by complex factors. Managing such a situation is important to prevent losses and injury. This study aimed to develop a dynamic model of human error assessment in emergencies in the petrochemical industry.

**Study design:** A cross-sectional study.

**Methods:** Fuzzy Bayesian network was used to improve the capabilities of the method for determining the control mode. Fuzzy-AHP-TOPSIS method was also used to prioritize emergency scenarios and human error assessment was applied for the most important emergency condition.

**Results:** Fire in a chemical storage unit was recognized as the most important emergency condition. Common Performance Conditions (CPCs) were determined based on the opinions of a panel of 30 experts and specialists and 7 CPCs were selected for emergencies; then, based on the results of AHP method the relative weights were determined. Finally, membership functions, inputs, and outputs of fuzzy sets, CPC values in 8 emergency response tasks, and the probability of control modes were determined using Bayesian Cognitive Reliability and Error Analysis Method (CREAM) method.

**Conclusion:** This method could be applied to overcome the weaknesses of traditional methods, provide a repeatable method for human error assessment, and manage human error in an emergency.

## Introduction

Emergencies are conditions, which may end to a range of effects such as mortality, disability-induced damage, destruction, pollution, damage to products, and equipment, or social consequences^1^. Emergency in the industry not only can result in a damage to critical infrastructure but also can lead to many consequences for industry workers, as well as other industry affiliates. Hence in recent years, theorists and experts have become more interested in managing dangerous conditions and reducing the damage caused by crisis and emergencies. Emergencies are unplanned and unpredictable circumstances associated with the lack of enough time to plan to deal with the problem, require excess physical resources and attempts to control the conditions, reduce cognitive performance,. It can cause stress in individuals^2^. The high level of long-term stress increases physiological changes in the human body, which in turn increases the risk of error in judgment or can cause operational errors. Therefore, due to stress, human performance in emergencies can also result in human error, and human error user such situations can lead to catastrophic consequences, including death, injury, disruption, psychological effects, as well as environmental impacts^3^.

In many major disasters, human error has been one of the main causes^4^. Therefore, human error analysis and appropriate control strategies are necessary to eliminate or reduce errors and negative consequences. Human error assessment methods are used to identify and systematically analyze and reduce the adverse effects of human error^3^. After the incidence of Three Mile Island disaster, this concept was introduced by experts and it was used as a quantitative and quantitative method to assess the contribution of human in accident. Human error also is the cause of many accidents in the petrochemical industry, and so far, many there has been many incidents caused by human error in this industry^5^. Human error studies started in the late 50's. These methods are generally divided into three categories. First-generation methods have been developed for the first time to assess risk and evaluate human error probabilities. These methods break the task into sub-tasks and then take into account the effects of factors such as time pressure, equipment design, and stress, to ultimately generate human error probability (HEP)^6^.

First-generation methods such as THERP and APJ focus more on skills and rules at a baseline levels and are criticized for ignoring underlying factors, organizational factors, and errors of the commission. However, first-generation methods still are used as a tool for quantitative risk assessment. Second-generation methods first were developed in 1990 based on cognitive models of human decisions and actions; in this group of methods, the quality of worker's performance depends on circumstances under which they work. Therefore, in the second-generation methods, organizational, technical, and human factors as well as expert judgment are involved in the evaluation of human errors ^7^. In these methods, factors that determine performance (PSFs or CPCs) are defined to assess the role of organization and environment on cognitive performance. Third-generation methods have been developed since 2005 and are used as a modeling and simulation system in the virtual environment. Bayesian models are among the third-generation methods used for human error evaluation^3^

Subjectivity and lack of data are the main drawbacks of human error assessment methods^8^. As another problem with human error assessment methods, they follow a binary (0 and 1) logic; as a consequence, evaluations does not well illustrate the effect of CPCs on the probability of human error, because human performance is influenced by complex and uncertain factors such as behavioral, psychological, and cognitive factors^9^. Accordingly, fuzzy sets are useful tools for modeling complex processes, when qualitative, inaccurate, and indeterminate data are used^8^.

Bayesian network (BN) is also a probabilistic tool used to describe relationships between variables while considering CPC dependencies. These networks are specific types of graphic models that represent the dependency between variables and can be used to make decisions under uncertainty ^8^. BN has been developed to provide specialized knowledge in fields where knowledge is uncertain, vague, or incomplete. Bayesian network includes graphical structures for representing possible relationships between large numbers of variables and making inferences out of those variables. Moreover, this tool could integrate qualitative variables with quantitative variables.

The aim of this study was to combine Bayesian networks (BN) and Fuzzy CREAM to develop a dynamic model for human error assessment in emergencies.

## Methods

### Select and prioritize emergencies

In order to select the emergencies and prioritize them, Delphi technique was used to extract the opinions of 12 experts and university professors and select important criteria in emergencies. In the next step, AHP method and fuzzy hierarchical analysis were used for paired comparison and to determine the weight and priority of selected criteria.

In the next step, a questionnaire with 11 options and 10 criteria was designed and sent to 30 safety and occupational health experts. They were asked to score each option based on the selected criteria and using linguistic scales to prioritize emergency situations according to the obtained scores.

### Human error assessment

Because of the complex nature of emergency tasks, there is always a risk of human error under such situations that may lead to a catastrophe for the entire organization. Therefore, it is necessary to pay attention to human errors in emergencies in terms of not only the probability but also the severity of the effects^3^.

CREAM is one of the most well-known methods used for identifying human error based on a psychological and cognitive model^10^. In this method, individual, organizational, and technical factors are considered as Common Performance Conditions (CPC) in 9 categories which include: adequacy of organization, working conditions, adequacy of MMI (Man and Machine Interface) and operational support, availability of procedures and plans, availability of time, time of day, adequacy of training and experience, and the quality of staff collaboration.

After identifying the tasks (using HTA method), initial screening of the individual's activities was performed and their effects (positive, negative or neutral) on the performance of individuals were assessed to determine control mode (Scrambled control, Opportunistic control, Tactical control, and Strategic control)^10^.

### CPCs adjustment

CPCs are dependent on and affect each other. To determine the impact of CPCs on performance, it is necessary to consider their dependency^3,10^.

### CPCs weighting in emergency situations

CPCs are different in various tasks and jobs and do not have the same effect on individuals' performance^11,12^; thus, Analytical Hierarchy Process and paired comparisons were used in this study to determine the relative importance of each CPC in emergencies. To this end, a pair comparison questionnaire was prepared and sent to 30 experts and specialists in the field of occupational safety and health.

The COCOM linguistic scale was determined based on the total weight of the CPC, using the following equation^13^.

$$C^{k}=\sum_{i=1,j=1}^{n}A_{i}.W_{j}$$

C^k^: Index for determining appropriate linguistic variables

A_i_: Relative weight of linguistic variable i

W_j_: Relative importance of the variable j

### Fuzzy CREAM

- Development of a fuzzy-CREAM classification method

Since uncertainty is one of the main problems in HRA, fuzzy method is proposed as a tool to solve this problem in CREAM method.

### Step 1: Selecting input variables

According to previous studies, CPCs used in various tasks and jobs are not the same and can be defined according to the type of task and job, and could be reduced or increased (modified by task). Therefore in this study, according to opinions of a panel of 30 experts and specialists in the field of safety and occupational health, and because of the lack of relationship between two CPCs, the number of CPCs was decreased to 7 CPCs^11,14^.

### Step 2: Determining fuzzy sets

Linguistic variables should be transformed into fuzzy and membership functions to better describe CPCs (positive, negative, and neutral). It is difficult to determine quantitative values for linguistic variables of CPCs. Fuzzy and mathematical rules are used to overcome this problem. According to previous studies, this method is effective to eliminate inaccuracy in human error assessment methods^3^. Due to the changes in linguistic variables of CPCs that increase, decrease, or remain neutral in an individual’s performance, their membership function was defined^4^.

### Step 3: Constructing a fuzzy rule base

COCOMs are discrete and definite while the intervals are large. To fix it, CPCs, as well as the control mode, should be converted to fuzzy values. COCOM linguistic scales for each rule were determined based on the total weight of the CPC and their weight, using the following relationship.

### Fuzzy sets determination

**Figure 1:**
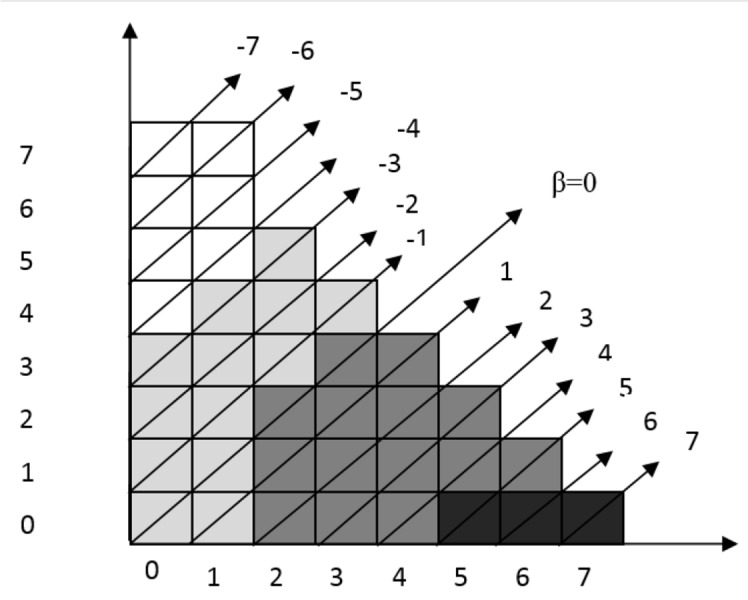
Determination of control mode based on context influence index

After determining the membership functions, using fuzzy if-then rules, inputs and outputs of fuzzy sets were determined using Figure 1 and the following relationships.

**Figure:**
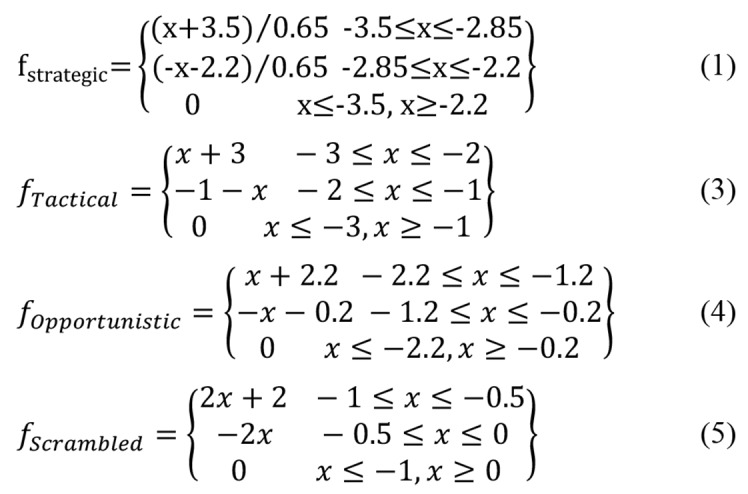


In general, Bayesian network is one of the data mining methods. There are three reasons highlighting the advantages of using this method. First, Bayesian network is a useful tool for incomplete data. Second, Bayesian network provides an opportunity to obtain knowledge on causal relationships. Third, Bayesian networks in combination with statistical methods can facilitate the integration of background knowledge and data^14^.

### Determine the probability of Bayesian networks

The basis of Bayesiannetwork calculations is Bayes' theorem. Bayes' theorem is based on the probability of an event, based on prior knowledge on related conditions and events. Based on Bayes' theorem, the probability of the incidence of an event is dependent on the occurrence of another event.

Bayes' theorem is stated mathematically as the following equation:

(6) $$P(B\left|A\right.)=\frac{P(A\left|B\right.)P(B)}{P\left(A\right)}$$

P(A) is the likelihood of event A

P(B) is the likelihood of event B

P(A│B) is the likelihood of event B, given that event A is true

A Bayesian network model has the following four steps:

- Definition of independent and dependent variables
- Establishing relationships between variables
- Defining variable modes
- Calculating the conditional probability for each node

In this study, Fuzzy CREAM was used as an input for Bayesian network. All calculations of BN were performed using GeNIe 2.0 software.

## Results

Prioritizing emergencies using Fuzzy TOPSIS method showed that fire in the chemical storage unit was the most important emergency situation; thus, human error assessment was applied for this emergency situation. CPCs were determined based on the opinions of a panel consisted of 30 experts and specialists in the field of safety and occupational health. The selected CPCs were: adequacy of organization, working conditions, adequacy of MMI and operational support, availability of procedures and plans, availability of time, adequacy of training and experience, and the quality of staff collaboration.

In addition, paired comparison of CPCs based on the AHP method was performed to determine their relative weights (Table 1). Adequacy of organization had the highest relative weight and adequacy of training and experience was ranked the second.

**Table 1:** Paired comparisons of the relative weight and Common Performance Conditions (CPCs)

**Common Performance Conditions**	**Relative weight**
Adequacy of organization	0.271
Working conditions	0.149
Adequacy of MMI and operational support	0.138
Availability of procedures and plans	0.122
Available time	0.064
Adequacy of training and experience	0.194
Crew collaboration quality	0.060

**Figure 2:**
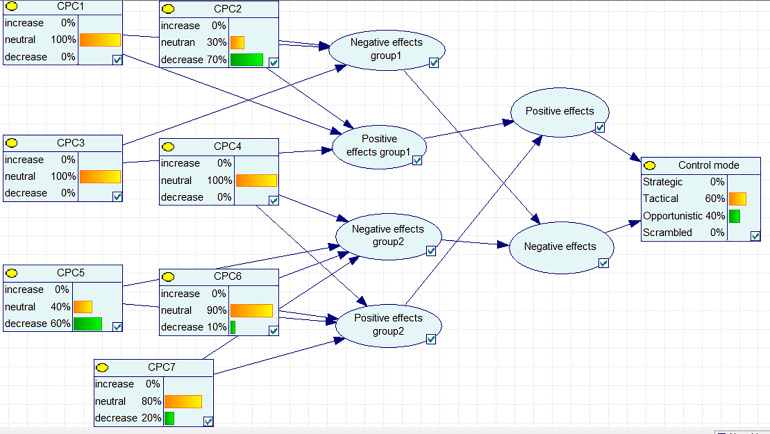
Bayesian network results for the coordination task

The data obtained on probability control modes are presented in Table 2 and Figure 2. Table 3 presents the data on the probabilities of weighted control modes.

**Table 2:** Probability of control modes

**Tasks**	**β**	**HEP**	**Crisp value**	**Strategic**	**Tactical %**	**Opportunistic %**	**Scrambled**
1	0.4	0.007	-2.1	0	85	15	0
2	1.0	0.009	-2.0	0	90	10	0
3	1.6	0.014	-1.8	0	60	40	0
4	1.4	0.012	-1.9	0	70	30	0
5	2.0	0.017	-1.7	0	55	45	0
6	1.2	0.011	-1.9	0	75	25	0
7	0.6	0.007	-2.1	0	90	10	0
8	0.3	0.006	-2.1	0	83	17	0

**Table 3:** Probabilities of weighted control mode

**Tasks**	**β**	**Weighted HEP**	**Crisp value**	**Strategic %**	**Tactical %**	**Opportunistic %**	**Scrambled**
1	0.11	0.006	-2.22	4	96	0	0
2	0.21	0.006	-2.19	0	99	1	0
3	0.17	0.006	-2.22	4	96	0	0
4	0.14	0.006	-2.21	2	98	0	0
5	0.30	0.006	-2.17	0	97	3	0
6	0.16	0.006	-2.21	2	98	0	0
7	0.11	0.005	-2.22	4	96	0	0
8	0.17	0.006	-2.20	0	100	0	0

## Discussion

The aim of this study was to develop a dynamic model for human error assessment in emergency situations. Given the unpredictability of human behavior, as well as the fact that human error is a natural part of human behavior^11^, one of the major challenges of industrial managers is to protect critical and vulnerable infrastructure at the time of accidents, emergencies, and crises.

The performance and response of individuals during accidents and emergencies have a very important role in reducing or increasing the risk. During an emergency, the reaction of individuals is affected by some factors such as stress, time deficiency, workload, etc.^11^, all of which increase the probability of human error. On the other hand, the probability of human error in emergency situations increases with time due to increase in fatigue^3^. Under such a condition, a mistake may lead to exacerbations of circumstances and uncontrollable damages and losses. The role of human error in major disasters in the world have also confirmed, for example, we may note London Beer Flood, Seveso, Bhopal, Chernobyl, Buncefield, and Tallmansville^11^.

Therefore, Human Reliability Analysis^15^ in emergencies is a very important tool to reduce the consequences of human error under such conditions. In this study, CREAM was used to evaluate HRA and human error. CREAM method is one of the second-generation HRA methods widely used in various industries including petrochemicals^12^. In this study, an applied three-step method was used to solve the weaknesses of this method. This three-step method included: determining CPC for emergency situations, determining the impact and relative weight of each CPC on the performance, and finally, the use of fuzzy and Bayesian rules to assess and reduce human error in such situations.

Human reliability is affected by individual, organizational, and environmental factors introduced in CREAM method under 9 CPCs. These CPCs are pre-defined and CPCs can be changed, increased, or decreased based on environment and working conditions. Eight modified CPCs were used for human error in tanker shipping^8^. Banda et al. also used 6 CPCs to evaluate human error in their study ^13^. In this study, in order to adapt CPC in emergencies, 7 CPC was used to assess human error based on opinions of experts and specialist; accordingly, membership relationships and fuzzy sets were defined based on these 7 CPCs.

As one of the weaknesses of CREAM method, the importance of CPCs in all jobs and tasks are considered equal, while CPCs do not have the same impact on human reliability and can take different weights depending on the type of tasks and jobs^16^. It is better to weight CPCs to show their real impact on human reliability^10^. To this end, various methods such as Delphi, pair-wise, and AHP can be used^17^. In this study, AHP method and paired comparison based on expert judgment were used to determine the weight of CPCs. Another important issue in human reliability assessment is the quantification of qualitative data on working conditions (based on expert judgments), as well as the ability to evaluate quantitative and qualitative data together^6^. CREAM method suffers from uncertainty problem, for instance uncertainty in input data and CPCs and uncertainty in control modes^8^. In recent years. in order to overcome the problems of uncertainty in decision making, uncertainty of complex issues, inaccurate information, and the combination of qualitative and quantitative data, fuzzy systems have been used as an appropriate method for solving such problems. In this study, a combination of fuzzy CREAM and Bayesian method was used to solve the mentioned problems. This method is a good tool for solving traditional CREAM problems^4,8,10,12,18-21^. The utilization of BN in human error assessment provides an effective mechanism for human reliability assessment under emergency conditions^21^. Bayesian network model was used to develop a basic CREAM method. Bayesian network was helped to overcome the weaknesses of CREAM method via reducing uncertainties in CPCs and led to instant and precise estimation of human error probability^18^.

In this study, the comparison of the results of control mode in normal and weighted items showed that the probability distribution of these two methods is different, and CPCs should be weighed in order to better reflect the working conditions. In this study, the worst scenario was related to Tasks 3 and 5, related to coordination tasks and pre-extinguishing operations. 'Coordination' is about coordination between different responding teams such as firefighting, rescue, HSE, medical, security, and public relations teams. On the other hand, 'pre-extinguishing operations' is elated to isolation of the area, control of volume, temperature, intensity of the fire, discharge, etc.

In these tasks, due to time limitation to control the situation and prevent the occurrence of a crisis, the probability of human error is higher, also reported and confirmed by previous studies^22^. Maneuvers and training to prepare to deal with this situation as well as the effectiveness of ERP is very important in preventing human error.

## Conclusion

Given the importance of identifying and controlling human error in emergencies, this study introduced and utilized an applied method to solve the weaknesses of traditional CREAM method and create a unique dynamic human error assessment method for each task. The results of this method can be used as a specific tool to manage human error in emergency. These results also can be utilized as a repeatable method for assessing human error in different industries and other critical jobs, depending to the conditions of the industry.

## Acknowledgements

This study has been adapted from a research project at Hamadan University of Medical Sciences. Thereby appreciate all contributed in this research.

## Conflict of interest

The authors declare that there is no conflict of interests.

## Funding

This study is supported by Hamadan University of Medical Sciences, Iran (grant No. 9611037026).

## Highlights

- Modifying the common performance conditions (CPCs) of CREAM method can better reflect the real situation.
- With considering input weights, outcomes are sensitive to alterations of input data and weights and producing more reliable outcomes.
- Combining Bayesian networks (BN) and Fuzzy CREAM in this method reduce the uncertainty in human error assessment.
- The suggested model makes it possible to instant calculation of human error probabilities.

## References

[1] Marwitz S, Maxson N, Koch B, Aukerman T, Cassidy J, Belonger D (2008). Corporate crisis management: Managing a major crisis in a chemical facility. J Hazard Mater.

[2] Paton D, Flin R (1999). Disaster stress: an emergency management perspective. Disaster Prev Manag.

[3] Petrillo A, Falcone D, De Felice F, Zomparelli F (2017). Development of a risk analysis model to evaluate human error in industrial plants and in critical infrastructures. Int J Disaster Risk Reduct.

[4] Karimie S, Mohammadfam I, Mirzaei Aliabadi M (2019). Human Errors Assessment in the one of the control rooms of a petrochemical industrial company using the extended CREAM method and BN. Saf Health Work.

[5] Jahangiri M, Hoboubi N, Rostamabadi A, Keshavarzi S, Hosseini AA (2016). Human error analysis in a permit to work system: a case study in a chemical plant. Saf Health Work.

[6] Marseguerra M, Zio E, Librizzi M (2006). Quantitative developments in the cognitive reliability and error analysis method (CREAM) for the assessment of human performance. Ann Nucl Energy.

[7] Kirwan B. A guide to practical human reliability assessment: CRC press; 2017.

[8] Zhou Q, Wong YD, Loh HS, Yuen KF (2018). A fuzzy and Bayesian network CREAM model for human reliability analysis–The case of tanker shipping. Saf Sci.

[9] Kim BJ, Bishu RR (2006). Uncertainty of human error and fuzzy approach to human reliability analysis. International Journal of Uncertainty Fuzziness and Knowledge-Based Systems.

[10] Yang Z, Bonsall S, Wall A, Wang J, Usman M (2013). A modified CREAM to human reliability quantification in marine engineering. Ocean Eng.

[11] Petrillo A, Zomparelli F. The importance of human error and reliability management in critical conditions and infrastructures. Human factors and reliability engineering for safety and security in critical infrastructures. Springer; 2018.

[12] Shirali GA, Hosseinzadeh T, Kalhori SRN (2019). Modifying a method for human reliability assessment based on CREAM-BN: A case study in control room of a petrochemical plant. MethodsX.

[13] Banda OAV, Goerlandt F, Kuzmin V, Kujala P, Montewka J (2016). Risk management model of winter navigation operations. Mar Pollut Bull.

[14] Bedford T, Bayley C, Revie M (2013). Screening, sensitivity, and uncertainty for the CREAM method of human reliability analysis. Reliability Engineering & System Safety.

[15] Aliabadi MM, Esmaeili R, Mohammadfam I, Ashrafi M (2019). Human reliability analysis (HRA) using standardized plant analysis risk-human (SPAR-H) and bayesian network (BN) for Pipeline inspection gauges (PIG) operation: A case study in a gas transmission plant. Health Scope.

[16] Hollnagel E. Cognitive reliability and error analysis method (CREAM): Elsevier; 1998.

[17] Wu B, Yan X, Wang Y, Soares CG (2017). An evidential reasoning‐based CREAM to human reliability analysis in maritime accident process. Risk Analysis.

[18] Yang Z, Wang J, Rochdi M, Belkacem O, editors. Bayesian modelling for human error probability analysis in CREAM. International Conference on Quality, Reliability, Risk, Maintenance, and Safety Engineering; 17 June -19 June: Xi'an, 2011.

[19] Ashrafi M, Davoudpour H, Khodakarami V (2017). A Bayesian network to ease knowledge acquisition of causal dependence in CREAM: application of recursive noisy‐OR gates. Quality and Reliability Engineering International.

[20] Mu L, Xiao B, Xue W, Yuan Z, editors. The prediction of human error probability based on Bayesian networks in the process of task. IEEE International Conference on Industrial Engineering and Engineering Management (IEEM); 6 Dec-9 Dec: Singapore, 2015.

[21] Musharraf M, Hassan J, Khan F, Veitch B, MacKinnon S, Imtiaz S (2013). Human reliability assessment during offshore emergency conditions. Saf Sci.

[22] DiMattia DG, Khan FI, Amyotte PR (2005). Determination of human error probabilities for offshore platform musters. J Loss Prev Process Ind.

